# Sobre los sentidos del cuidado: Narrativas de adolescentes con experiencia de vida en situación de calle en la Ciudad Autónoma de Buenos Aires, Argentina

**DOI:** 10.18294/sc.2025.5401

**Published:** 2025-04-11

**Authors:** María Sol Bezzecchi, Carolina Guerrero, Melisa Scampini, Paula Marcela Albornoz, María Malena Lenta

**Affiliations:** 1 Licenciada en Trabajo Social, Especialista en Salud Mental Comunitaria. Trabajadora Social, Hospital Tobar García, Centro Barrial Barracas, Vientos de Libertad, Movimiento de Trabajadores Excluidos. Ciudad Autónoma de Buenos Aires, Argentina. mariasolbezzecchi@gmail.com Hospital Tobar García Hospital Tobar García Ciudad Autónoma de Buenos Aires Argentina mariasolbezzecchi@gmail.com; 2 Licenciada en Terapia Ocupacional. Especialista en Salud Mental Comunitaria. Residente, Residencia Postbásica de Investigación en Salud Publica y Epidemiologia, Hospital Interzonal General de Agudos San Roque, La Plata, Buenos Aires, Argentina. to.guerrerocarolina@gmail.com Hospital Interzonal General de Agudos San Roque Residencia Postbásica de Investigación en Salud Publica y Epidemiologia Hospital Interzonal General de Agudos San Roque La Plata Buenos Aires Argentina to.guerrerocarolina@gmail.com; 3 Licenciada en Trabajo Social. Especialista en Salud Mental Comunitaria. Becaria, Hospital Nacional de Pediatría “Prof. Dr. Juan P. Garrahan”. Ciudad Autónoma de Buenos Aires, Argentina. scampinimelisa@gmail.com Hospital de Pediatría SAMIC Prof. Dr. Juan Pedro Garrahan Hospital Nacional de Pediatría “Prof. Dr. Juan P. Garrahan” Ciudad Autónoma de Buenos Aires Argentina scampinimelisa@gmail.com; 4 Enfermera Profesional. Especialista en Salud Mental Comunitaria. Enfermera, Instituto Superior de Otorrinolaringología, Ciudad Autónoma de Buenos Aires, Argentina. Paumar1979@gmail.com Instituto Superior de Otorrinolaringología Instituto Superior de Otorrinolaringología Ciudad Autónoma de Buenos Aires Argentina Paumar1979@gmail.com; 5 Doctora en Psicología. Becaria Posdoctoral, Consejo Nacional de Investigaciones Científicas y Técnicas. Docente-investigadora, Universidad de Buenos Aires, Ciudad Autónoma de Buenos Aires, Argentina. malenalenta@gmail.com Consejo Nacional de Investigaciones Científicas y Técnicas Consejo Nacional de Investigaciones Científicas y Técnicas Universidad de Buenos Aires Ciudad Autónoma de Buenos Aires Argentina malenalenta@gmail.com

**Keywords:** Modelos de Cuidado, Adolescentes en Situación de Calle, Argentina, Healthcare Models, Adolescents Experiencing Homelessness, Argentina

## Abstract

La micropolítica del cuidado permite entender su construcción de manera multívoca en las interacciones cotidianas, la negociación de significados y el modo en que las personas se organizan para sostener la vida, incluso en contextos de vulneración de derechos. El objetivo del trabajo es explorar los sentidos sobre el cuidado que construyen adolescentes institucionalizados en situación de calle, en la Ciudad Autónoma de Buenos Aires en el periodo octubre-diciembre de 2023. Se realizó una investigación cualitativa, con aportes de la investigación acción participativa, con ocho adolescentes varones en dos hogares convivenciales. Por medio de entrevistas en profundidad, grupos focales y observaciones participantes, se abordaron los diversos sentidos del cuidado, vinculados a sus experiencias de vida. Entre sus vínculos afectivos mencionan la familia, las instituciones de alojamiento y sus pares/amigos. Las estrategias de cuidado identificadas son rescatar(se), hacer uso de instituciones y hablarle a un otre. En las conclusiones, se propone la centralidad de la categoría cuidado, de la cual se desprenden los sentidos de los vínculos afectivos y las estrategias de cuidado, y se plantean interrogantes para repensar formas de acompañar adolescencias como profesionales de salud.

## Abstract (en)

The micropolitics of care allows for an understanding of its construction as multifaceted, shaped through everyday interactions, negotiation of meanings, and the ways in which people organize themselves to sustain life, even in contexts where rights are being violated. The aim of this study is to explore the meanings of care constructed by institutionalized adolescents experiencing homelessness in the Autonomous City of Buenos Aires between October and December 2023. A qualitative study was conducted, incorporating elements of participatory action research, with eight male adolescents living in two residential care homes. Through in-depth interviews, focus groups, and participant observation, the study addressed the various meanings of care as related to their life experiences. Among their affective bonds, they mentioned family, residential institutions, and peers/friends. The identified care strategies include rescuing themselves, making use of institutions, and speaking to an “other.” In the conclusions, the centrality of the category of care is proposed, from which the meanings of affective bonds and care strategies emerge, and questions are raised to rethink how health professionals can support adolescents.

## INTRODUCCIÓN

La cuestión de las adolescencias que habitan las calles se configura como una problemática social compleja, tanto en nuestro país como en otros países de Latinoamérica. La (in)visibilidad que se le da debe ser leída en clave epocal y contextual, enlazada a las políticas destinadas a su abordaje^1^.

En este sentido, el marco normativo actual vinculado a la restitución de derechos de las niñeces y adolescencias (Convención sobre los Derechos de Niños, Niñas y Adolescentes de 1989; Ley 144 de 1998 de Protección Integral de los Derechos de Niños, Niñas y Adolescentes de la Ciudad Autónoma de Buenos Aires; Ley 26061 de 2005 de Protección Integral de los Derechos de Niñas, Niños y Adolescentes), tiene por objetivo proteger a las niñeces y adolescencias para que puedan disfrutar y ejercer en forma plena y permanente todos los derechos reconocidos por las normas nacionales y los tratados internacionales de derechos humanos. La Ley de Protección Integral, en tanto dispositivo cultural, técnico y jurídico-legal, habilita la intervención del Estado, bajo la forma de prestación de servicios especializados, que se diferenciarían de los principios caritativos de la esfera privada^2^.

Se incorpora el principio del interés superior de la niñez y la obligación del gobierno de adoptar las medidas legislativas, administrativas y de otra índole para dar efectividad a los derechos. Por lo tanto, priorizar la opinión de las niñeces y adolescencias es un punto de partida y una posición ético-política sustentada en un marco legal y normativo. Sin embargo, en la Ciudad Autónoma de Buenos Aires, no se dan instancias para que las voces de las niñeces y adolescencias sean consideradas al momento de la planificación, ejecución y evaluación de las políticas públicas. A su vez, la universalización y enunciación de los derechos no iguala las condiciones de posibilidad para el acceso integral a los derechos de cada niñe o adolescente, el cual se ve atravesado por diversas intersecciones^3^.

En este sentido, la ciudadanía de la infancia no es una condición dada por la propia existencia de niñeces y adolescencias, como personas concretas, sino que debe ser considerada en la tensión entre la heteronomía y la autonomía, y depende de la vida política para poder generar las condiciones de puesta en ejercicio y de exigibilidad^4^. Las condiciones de vida actuales, signadas por la precarización de la vida, la vulneración de derechos, los obstáculos en el armado y sostén de lazos y la falta de soportes comunitarios exacerban las dificultades para apuntalar las trayectorias de vida de las niñeces y adolescencias inscriptas en el contexto actual. Es así que un sector de las niñeces y adolescencias se vuelve poblador de territorios amenazantes. Al mismo tiempo, las condiciones de vida precarias reducen las expectativas de futuro a la supervivencia del día a día, configurando situaciones y territorios de vulnerabilidad psicosocial^5^.

Asimismo, el contexto actual de expulsión y ampliación de la pobreza generó que un vasto sector de las niñeces y adolescencias se encuentren en situación de vulnerabilidad psicosocial. Tal situación, definida desde la lógica del déficit, genera las condiciones necesarias para que la institucionalización continúe siendo la modalidad privilegiada de protección, pese a la existencia de reglamentaciones que enuncian lo contrario^6^. La institucionalización por causas asistenciales, como parte de los procesos de tutelarización o de minorización, produce un tipo de subjetividad particular, tradicionalmente abordada desde la perspectiva hegemónica de la normalización, que reproduce la idea de una única niñez posible^5^.

En contraposición, entender a las niñeces y adolescencias como sujetos políticos, que tienen una posición activa para pensar sus propios procesos de vida, implica contemplar la corresponsabilidad del conjunto de las instituciones sociales en el apuntalamiento de la autonomía progresiva^7^. Por lo que aprender sobre sus estrategias y vivencias posibilita pensar el accionar como actores del sistema de salud, de manera más situada y crítica. A su vez, creemos que trabajar desde la potencia, el deseo y lo posible le permite a las adolescencias posicionarse desde otro lugar, que recupera los saberes y experiencias propias frente a las perspectivas pasivisantes de las políticas tutelares y minorizantes.

En este marco, el objetivo de este trabajo es explorar los sentidos sobre el cuidado y las redes afectivas y vinculares que han co-construido en sus trayectorias de vida los adolescentes con experiencia de vida en calle, que participan en dos instituciones de alojamiento del Sistema de Protección Integral de Derechos de Niñas, Niños y Adolescentes: el Centro de Atención Integral para la Adolescencia y la Niñez (CAINA) y La Boquita, ambos situados en la Ciudad Autónoma de Buenos Aires.

El propósito del estudio es reconocer diversas categorías que las adolescencias participantes de dichas instituciones producen en su vida cotidiana desde la propia perspectiva juvenil. Desde esta perspectiva, se propone eludir la mirada adultocéntrica, entendida como una relación social basada en la centralidad de “lo adulto”, que conforma un sistema de poder asimétrico, el cual legitima algunos preconceptos vinculados a las niñeces y adolescencias^8^.

Las instituciones en las que realizamos nuestro trabajo de campo forman parte de la política pública del Gobierno de la Ciudad Autónoma de Buenos Aires, que pretende abordar las niñeces y adolescencias que habitan las calles. Hablamos de habitantes porque, como señala Paula Cecilia Rosa:

…se entiende que estos habitan el espacio de la calle, pues allí entablan una relación con el entorno (se apropian y hacen uso de este espacio) y establecen vínculos e interacciones con diferentes personas o grupos que se encuentran en su misma situación o no (vecinos, comerciantes, transeúntes, etc.)^9^

Los resultados del Relevamiento Nacional de Personas en Situación de Calle (ReNaCALLE)^10^, realizado en diciembre de 2023, arrojaron que 1.104 niñas, niños y adolescentes (menores de 18 años) se encuentran en situación de calle en nuestro país. Cabe mencionar que estos datos surgen de niñas, niños y adolescentes que se encuentran con personas adultas que fueron censadas y lo informaron, ya que no se entrevistaron a quienes se encontraban sin compañía de una persona adulta referente al momento del censo. A su vez, el Censo Nacional de la República Argentina de 2022^11^ tampoco informa datos sobre las niñeces y adolescencias en situación de calle por el mismo motivo. Asimismo, según el informe “Situación de niñas, niños y adolescentes sin cuidados parentales en la República Argentina” presentado por la Secretaría de Niñez, Adolescencia y Familia (SENAF), a finales de 2020 había más de 9.000 personas menores de edad sin cuidados parentales, alojados en dispositivos de cuidado residencial o familiar, aunque no se indica en el informe si han tenido o no experiencia de vida en calle^12^.

Sin embargo, a pesar de la falta de datos precisos sobre niñeces y adolescencias con experiencia de vida en calle, al momento de la realización de la investigación, la Ciudad Autónoma de Buenos Aires cuenta con políticas específicas para este sector de la población en situación de calle que hacen parte del sistema de protección de derechos de niños, niñas y adolescentes en dicha jurisdicción. Estos dispositivos dependen del Consejo de los Derechos de Niñas, Niños y Adolescentes desde mayo de 2021, y pertenecían anteriormente a la Dirección General de Niñez y Adolescencia del Ministerio de Desarrollo Humano y Hábitat de la Ciudad. Estos dispositivos corresponden a tres centros de día y noche (establecimientos convivenciales que proporcionan un lugar de residencia que garantiza la satisfacción de las necesidades básicas de protección y el ejercicio de derechos de niñeces y adolescencias que habitan las calles) de los cuales dos son para adolescentes varones (CAINA y La Boquita) y uno para mujeres (Hogar Nuestra Señora de Guadalupe). Asimismo, la Ciudad dispone de *hogares convivenciales conveniados* (establecimientos de gestión asociada con organizaciones no gubernamentales que proporcionan un lugar de residencia que garantiza la satisfacción de las necesidades básicas de protección y el ejercicio de los derechos de niñas, niños y adolescentes que precisan, de manera transitoria, un contexto de convivencia alternativo a su familia de origen); *comunidades terapéuticas conveniadas* (dispositivos residenciales de gestión asociada para el abordaje de consumos problemáticos); y el *equipo móvil de abordaje territorial* (equipos de profesionales que desarrollan un servicio de detección y contención a niñeces y adolescencias que habitan las calles).

La dinámica cotidiana actual de La Boquita y el CAINA se caracteriza por la posibilidad de permanencia en la institución. Allí desarrollan sus actividades diarias como alimentación, descanso o higiene. Estas son mediadas por vínculos, afectos, conflictos y cuidados que se dan entre adolescentes, así como con las personas trabajadoras de la institución. En cuanto al ingreso de adolescentes al dispositivo, es de manera voluntaria, o por derivación del Centro de Admisión y Derivación (CAD) o de medidas judiciales de protección, aunque no son excluyentes. Una vez que los adolescentes ingresan, el equipo de trabajadores comienza un proceso de acompañamiento integral y singular. En este sentido, se intenta priorizar la revinculación familiar y/o con referentes afectivos (Registro de campo, 2023).

Partimos de pensar a las adolescencias en plural, entendiendo que en cada momento histórico podemos observar múltiples y diversas formas de adolescencia fuertemente condicionadas por el contexto de vida y las condiciones de existencia. Entendemos que dicho concepto es un constructo histórico, que responde a la cronologización de la vida, que viene acompañada de la institucionalización del curso de vida a través de la intervención del Estado^13^.

En este sentido, se van conformando políticas públicas y sociales que buscan potenciar y acompañar, o vigilar y estigmatizar, dependiendo muchas veces de los atravesamientos socioeconómicos, siendo en las adolescencias que habitan las calles en quienes se concentran históricamente las mayores intervenciones destinadas a normalizar y normativizar aquello que se corre de lo esperado^14^.

Las adolescencias son habladas y nombradas por otras personas, inscribiéndose en ellas diferentes imágenes: atrevidas, malvenidas, peligrosas. Se las presupone mudas, sin embargo, también se espera que puedan poner en palabras cuando se las intenta interpelar, requiriendo que puedan encarnar aquellos roles asignados previamente. Quizás sus silencios son una invitación a escuchar un murmullo, a darle lugar a lo no dicho^15^.

Esta lectura estandarizada y normativa se refleja también en las formas de entender los cuidados, inscribiéndose como prácticas homogéneas e iguales para todas las personas y, por tanto, que invisibilizan las diversas condiciones de vida que atraviesa cada sujeto. No llamamos cuidados a los gestos de atención al otro, desprovistos de respeto y escucha sobre sus necesidades, deseos, posibilidades y creencias^16^. Por lo contrario, entendemos que existe una producción subjetiva de los cuidados, es decir, las formas de cuidar no devienen estandarizadas, sino que responden a la singularidad entre quien cuida y a quién se dirigen esos gestos de cuidado^17^. Esto permite pensar los cuidados mediados por las condiciones de posibilidad singulares y colectivas, las significaciones construidas en su entorno y las dimensiones contextuales que hacen a las condiciones de ser/estar en el mundo. Incluso, cuando las condiciones de posibilidad están atravesadas por múltiples vulneraciones y violencias, coexisten otras formas de producir cuidados y sentidos. De este modo, intentamos explorar la producción de cuidado entre adolescentes como

…puntos de fuga a lo normativo, [...] un cuidado entendido, sobre todo, como una producción de existencia en la otra persona, desde un posicionamiento político comprometido que contempla el derecho a la diferencia.^18^

## METODOLOGÍA

La investigación se realizó en el marco de la Residencia Interdisciplinaria de Salud Mental (RISaM) del Hospital Nacional en Red “Lic. Laura Bonaparte”. En función del problema planteado, se propuso un estudio con un enfoque cualitativo, ya que “proporciona profundidad a los datos, dispersión, riqueza interpretativa, contextualización del ambiente o entorno, detalles y experiencias únicas”^19^. Este enfoque metodológico comprende que la realidad es subjetiva, y se construye entre las personas investigadoras y las que participan. En este sentido, busca tener una mirada situada sobre los fenómenos estudiados, sus múltiples relaciones y contradicciones y en el contexto en que se inscriben.

El diseño fue de tipo exploratorio con componentes descriptivos, con una modalidad abierta, flexible y prospectiva. Asimismo, el método se encuentra orientado al proceso*,* y no al resultado^18^, ya que sobre todo nos interesó poder hacer partícipes a los adolescentes en las distintas etapas de la investigación. En este sentido, se tomaron elementos de la estrategia de investigación de acción participativa. El componente “participativo”^19^ le proporciona el rasgo característico a esta estrategia, dado que parte de considerar a las adolescencias como participantes activos, por tal motivo sus “voces” resultan esenciales durante todo el proceso. Asimismo, esta estrategia implica entender la investigación como parte de la práctica y como acción transformadora.

Para la realización del estudio se organizó una muestra de tipo intencional no probabilística por conveniencia, de casos típicos, oportunos y accesibles. La misma quedó conformada por ocho adolescentes que se autopercibían varones cis-género de entre 14 y 18 que concurrían a La Boquita y el CAINA durante el periodo octubre-diciembre de 2023. Como criterios de inclusión/exclusión se tuvo en cuenta la voluntad de participar y la situación de salud integral del adolescente, siendo excluidos aquellos casos en los que el equipo técnico de las instituciones sugirió la no participación en el estudio.

Las herramientas para la producción de datos fueron observación participante, grupos focales y entrevistas semiestructuradas que se implementaron con los adolescentes participantes. Asimismo, al inicio del estudio se realizaron entrevistas a informantes claves (referentes y trabajadores de las instituciones), con el objetivo de generar un primer acercamiento, conocer las dinámicas institucionales y características de los jóvenes.

La observación participante tuvo por objetivo conocer aspectos de la vida cotidiana de los adolescentes y comprender de manera situada las vinculaciones con su contexto. Esta herramienta comenzó a implementarse al inicio del trabajo de campo y permitió generar un primer acercamiento a su cotidiano, así como construir vínculos de confianza desde la horizontalidad. La observación participante permitió construir conceptos sobre situaciones sensibles, en las que se puede encontrar dificultad para discutir o describir^19^. Durante estas observaciones se utilizaron registros en diarios de campo, que incluyen las interpretaciones y disposiciones de los participantes.

También se implementaron cinco grupos focales de aproximadamente tres horas de duración. Esta técnica fue pertinente para recuperar y elaborar representaciones, experiencias, sentimientos, entre otros, de manera grupal^20^; y permitió aprehender y construir categorías desde el intercambio entre los adolescentes. En este sentido, durante el trabajo, enfatizamos sobre la importancia de poder tomar otros registros que no tuvieran que ver únicamente con la palabra hablada, ya que muchas veces nos transmitieron sentires y afectos de otros modos, con gestos corporales, bromas, miradas. A su vez, evitó caer en la individualización de aspectos sociales e históricos, permitiendo tensionarlos y ponerlos en diálogo.

Finalmente, en la fase final del trabajo de campo, se realizaron cuatro entrevistas semiestructuradas con los adolescentes, de forma individual, que permitieron profundizar la producción de sentidos sobre situaciones que no emergieron en instancias grupales. Asimismo, ofrecieron otro marco de intimidad que posibilitó conocer aspectos de sus trayectorias de vida. Dichas entrevistas fueron grabadas en aquellos casos en los que los adolescentes prestaron su consentimiento oral para ello.

Para el análisis de datos, en un primer momento, se realizó la transcripción de los registros grabados y/o escritos a mano en el cuaderno de campo. En un segundo momento, se realizó un análisis de contenido temático^21^. Con el apoyo de una grilla de sistematización se realizó una lectura horizontal y luego otra transversal de la información, lo que permitió definir categorías y temas.

Con relación a las consideraciones éticas, el estudio contó con la aprobación del Comité de Ética del Hospital Nacional en Red Especializado en Salud Mental y Adicciones “Licenciada Laura Bonaparte” [IF-2023-111064811-APN-HNRESMYA#MS]. También se suscribió un consentimiento informado institucional y se trabajó el consentimiento oral y el asentimiento de la participación de los adolescentes en diferentes momentos del proceso de investigación. Este último refiere a un “proceso relacional a partir del cual quienes participan expresan tanto de modo no verbal como verbal su voluntariedad de seguir participando’’^22^. Asimismo, tanto en las dinámicas grupales como en las entrevistas individuales se resaltó y reforzó sobre su derecho a no participar o a retirarse en cualquier momento. Finalmente se destaca que los nombres de los adolescentes participantes han sido modificados para resguardar su intimidad.

## RESULTADOS

Para la presentación de los hallazgos del estudio, partimos de identificar los sentidos producidos en torno a la categoría cuidado que se desprenden de las narrativas de los adolescentes, lo que implica identificar a los objetos de cuidado, en estrecha vinculación con las experiencias de vida de estos adolescentes.

En un segundo momento, derivado del análisis de los sentidos producidos en torno al cuidado, nos adentraremos en la concepción de vínculos afectivos que se desprenden de la relaciones entre las personas imbricadas en el cuidado. Compartimos coordenadas en torno a la relación entre los vínculos construidos y los gestos de cuidado^16^ recibidos, así como aquello que vuelve significativos a esos vínculos, desde la perspectiva de los adolescentes.

En un tercer momento, se analizan las estrategias de cuidado de los adolescentes, es decir, aquellas formas de cuidarse y cuidar que co-construyeron en sus vidas cotidianas.

### Los sentidos sobre el cuidado

Al abordar los sentidos sobre el cuidado, las narrativas dan cuenta de dos sentidos que se conectan y complementan:

E: *Para vos, ¿qué es cuidar algo o alguien?*

Bauti: *Cuidarlo… acompañarlo, fijarse lo que necesita y no sé qué más…* (Entrevista 3, 2023)

Eze: *Cuidar sería que no te dejen salir a la noche… porque capaz que hay zonas complicadas… te pueden robar, te pueden hacer algo por un celular que no vale la pena… Y eso sería cuidar, ir al médico, todas esas cosas que yo creo, ¿no? Cuando no ves mucho, no poder usar la bicicleta, que no te dejan… eso también es cuidado, ¿no?* (Entrevista 5, 2023)

E: *¿Cómo era cuando cuidabas a tu hermanita?, ¿qué hacías? Samuel: no la cuidaba, la miraba… No, mentira, cuidaba cuando... ponele que cuando ella pide algo, me pide a mí.* (Entrevista 4, 2023)

En los relatos aparece el cuidado en el sentido de estar atento a las necesidades de una otra persona o que otra persona esté atenta a sus necesidades. El miramiento *“*mirar con amoroso interés a quien se reconoce como sujeto ajeno y distinto de uno mismo”^23^, el acompañar y estar disponible a esa otra persona, son características que atraviesan los cuidados definidos por ellos.

Estos sentidos atribuidos al cuidado implican acompañar desde la singularidad de a quién se cuida, conocerla, llevar adelante acciones, dar consejos e incluso marcar límites en función de las particularidades y condiciones de vida. En este sentido, podríamos vincular las narrativas con el concepto de interdependencia que proponen los transfeminismos, en tanto crítica a la individualización y autodeterminación de las vidas. Esta se encuentra acompañada de la idea de entrelazamiento “para vivir dependemos de otres y pares dependen de nosotres. La vida de cada une está enlazada [...] a la vida de otres”^24^.

La interdependencia implica el reconocimiento de la vulnerabilidad compartida, de saber que todas las personas somos sujetos vulnerables y desde allí se habilitan y recepcionan algunas prácticas y gestos de cuidado. Cabe aclarar que el reconocimiento de la vulnerabilidad compartida presenta contradicciones, circulando a la par formas de nombrarse desde el poder en soledad. Se entiende que ambos aspectos conviven y coexisten en las formas en las que se define el cuidado. Estas formas de significarlo se distancian de concepciones que entienden el cuidado como prácticas estandarizadas, homogéneas e iguales para todas las personas y, por tanto, que invisibilizan las diversas condiciones de vida que atraviesa cada sujeto^16^.

Asimismo, los sentidos sobre el cuidado no están desprovistos de la consideración de los objetos de cuidado, es decir, aquello a lo que estos adolescentes nombran como lo que “hay que cuidar”, lo cual se encuentra en estrecha relación con aquello de lo que “hay que cuidarse”.

En las narrativas insiste el sentido de “lo propio” como aquello que “hay que cuidar”, pudiendo tomar diversas formas. Por un lado, puede entenderse enlazado al cuidado del cuerpo:

E: *Y ¿de qué pensás que es importante cuidarse?*

Bauti: *¿Cuidarse? Y yo qué sé, de las enfermedades y… no sé… de meterte en alguna droga que no tenés que consumirla*. (Entrevista 3, 2023)

Eze: *Es importante* [cuidar] *la salud, de todo eso, de los choques, bueno yo ya tengo cada choque. Cada palo tengo* […] *Es mejor que me cuiden porque vos no sabés con quién te podés encontrar en la calle. Con qué gente mala*. (Entrevista 5, 2023)

En el sentido de las narrativas, el cuerpo aparece como aquello que hay que cuidar frente a lo que puede dañarlo, como enfermedades, el consumo de sustancias, accidentes, eventos o personas. Por sus historias y experiencias previas, surge el sentido de que es necesario “*cuidarse de*” estar expuestos a zonas que implican riesgos y personas que podrían ocasionarles algún daño. Asimismo, a medida que avanzan en sus relatos, aparecen otras complejidades y concepciones acerca de “lo propio”, lo que puede tomar otras formas como objetos y personas de confianza para ellos:

E: *¿Qué te parece que es importante cuidar?*

Bauti: *¿Cuidar? Y no sé. Como lo de uno propio…*

E: *¿Cómo tus cosas o como personas?*

Bauti: *Los dos.* (Entrevista 3, 2023)

Bauti: *Pero al Nacho le bajé una raya hoy también. Se quiso adueñar de mi visera, pero no lo dejé.* (Entrevista 3, 2023)

En lo desarrollado se visibiliza la coexistencia de dos dimensiones. Por un lado, una concepción y significación del cuidado en tanto estar para otras personas y que otras estén para ellos. Por otro lado, los objetos de cuidado que en términos generales son mayoritariamente mencionados desde la individualidad y la propiedad de cada uno. En este sentido en los relatos se entrecruzan aspectos que dan cuenta de la construcción de subjetividades situadas en un escenario social contemporáneo, donde prima cierto individualismo y en donde les toca enfrentar constantemente situaciones de violencia que los vuelve, al mi*s*mo tiempo, cuidador y propietario de la propia vida (“*nadie te va a cuidar por vos*”)^15^.

Algunos autores^15^ conceptualizan sobre la precariedad totalitaria de las vidas, cuando todo lo que se arma para vivir (relaciones, amores, consumo) se sustenta sobre esta precariedad. Desde este suelo, cualquier roce puede generar situaciones que desequilibran o afecten el cotidiano. Las trayectorias en calle no solo permean de lo que hay que cuidarse, sino también en los objetos de cuidado porque hay que cuidar individualmente lo que se tiene, porque muchas veces no hay dónde resguardarse y menos aún resguardar aquello que se considera propio.

Ante esto, nociones como “*bajar raya*”, entendida como marcar un límite o impedir una acción que los perjudica, e insistir sobre la propiedad privada y lo propio (signo también de la época) puede ser una respuesta posible para resguardar aquellos objetos y vínculos que son parte, hacen y sostienen a sus cotidianos.

Esta tensión, entre la interdependencia y lo insistente de lo propio y lo privado, a su vez dialoga con la insistencia de que para cuidarse hay que “poder solo”, resonando formas de nombrarse en soledad:

Samuel: *Me cuido solo, me banco yo solo* [golpeando el puño contra su otra mano]. (Entrevista 4, 2023)

E: *Sí, o pedir que le pongan más un límite capaz, o que te cuiden más.*

Facu: *Yo me cuido solo*. (Entrevista 1, 2023)

Si bien no se nace en soledad, se llega a estarlo^25^. Las narrativas hablan de las propias historias, de sentimientos de soledad, de no haber recibido cuidados, de haber tenido que hacerse cargo de hermanas y hermanos, de tener que habitar la calle como salida ante la falta de cuidados adultos, entre otros. Esto se inscribe en una operatoria política del neoliberalismo, que exige la autosuficiencia, dejando por fuera los diferentes puntos de partida sorteados en un sistema desigual, patriarcal y capacitista, que (re)produce la individualización de las vidas.

Si bien estas lógicas se inscriben en las diversas identidades de género, por constituir una de las bases de la estructura social actual, es importante mencionar que se configura de manera singular en las masculinidades normadas. En este punto, resulta pertinente hacer una lectura enmarcada en los mandatos y roles que simbolizan varones cis. De ellos se espera que reproduzcan los valores socialmente construidos y legitimados de autosuficiencia, valentía y fortaleza para enfrentar no solo sus cotidianos, sino también los riesgos y conflictos a los que están expuestos^25^.

El “poder solo” se enmarca en contextos hostiles y signados por múltiples violencias (policial, entre pares, institucional, familiar) donde tal vez no hubo otra persona presente para ejercer cuidados y alojar. Por eso, la premisa de “poder solo”, también puede ser una respuesta o salida frente aquello que no se encontró en sus trayectorias de vida. Pues la lógica de que “si nadie me cuida entonces me cuido yo solo”, en definitiva, tiene un correlato con la realidad en la que tuvieron que escapar, dormir en la calle y defenderse solos, “poder solo”:

*Víctor le dice a Coco que no tiene calle, ante lo cual Coco le muestra una cicatriz respondiendo que eso es por la calle. Le cuenta que fue por una pelea y que se curó solo. Dijo que en general quienes están en la calle no van al hospital, no porque no les atienden bien, sino porque no creen que eso sea para ellos, porque pueden solos*. (Registro de campo, 2023)

Que los adolescentes que habitan la calle crean que un centro de salud no es para ellos también da cuenta de las dificultades del Estado, materializado en personas concretas que lo representan, para dar respuestas y generar condiciones de accesibilidad. La práctica en salud que menciona Coco, en la que pareciera que “lo atienden bien”, garantiza la atención, pero no el real acceso. Este no sentir el sistema de salud como un lugar al cual poder ir, habla de la necesidad y responsabilidad en tanto Estado y trabajadores de la salud, de desanudar ambos sentires. Este nudo probablemente tenga un correlato con historias en las que no han sido recibidos como los pacientes esperados.

Estos no-movimientos y no salir en búsqueda de aquellos adolescentes que se encuentran padeciendo en soledad, no solo afectan el acceso a dispositivos de salud, sino que insisten sobre la responsabilidad del cuidado de ellos mismos, ubicando por acción u omisión qué cuerpos pueden concurrir a ciertos espacios y cuáles no.

En un contexto marcado por tecnologías productivas de precarización afectiva, la soledad se presenta como mérito de “poder solo’’. No obstante, los vínculos afectivos junto con ciertos gestos de cuidados que los constituyen están presentes también en las narrativas.

### Vínculos afectivos

A continuación, se presentan aquellos vínculos afectivos que fueron significados en torno al cuidado como relevantes, lo que incluye su presencia efectiva pero también su ausencia o anhelo. Esto vínculos fueron aquellos relacionados con la familia nuclear, con las instituciones La Boquita y el CAINA y sus referentes, y con los pares y amigos.

#### Vínculo con su familia nuclear

Las personas de la familia son nombradas al indagar en torno al cuidado, tanto por su ausencia como por haber mantenido encuentros y vincularse en momentos puntuales de sus trayectorias de vida. Quienes aparecen como cuidadores son tías y tíos, abuelas y abuelos y hermanas y hermanos. Se destaca de estos últimos el cuidado mutuo en momentos en los que se encontraban “solos”, es decir, sin el cuidado de personas adultas:

E: *Vos también los cuidabas* [a los hermanos]

Bauti: *Y sí, cuando podía*…

E: *¿Y por qué pensás que se cuidaban?*

Bauti: *No sé, nosotros vivimos solos desde hace un tiempo, y siempre la pasamos juntos, trabajamos juntos, vivimos juntos, hicimos todo juntos y…* [silencio]. (Entrevista 3, 2023)

E: *¿En qué lugares te sentiste cuidado a lo largo de tu vida o qué personas te cuidaron?*

Facu: *Mi familia.*

E: *¿Y por qué creés que te cuidan?*

Facu: *Porque sí, de chiquito la pase re mal.*

E: *¿Cómo estaba conformada tu familia?*

Facu: *Por mi familia... mis tíos, mi abuela*. (Entrevista 1, 2023)

E: *Viste que a fin de año, a veces se piden deseos para empezar el año, ¿vos pensaste en alguno?*

Facu: *Sí, en seguir teniendo la familia mejor*. (Entrevista 1, 2023)

En las narrativas se destaca una concepción de familia ligada a las ideas tradicionales que se establecen como “familia”. Desde esta concepción, lo que la constituye es la existencia de un lazo que, en principio, no es elegido, sino que viene dado por el parentesco. En sus narrativas, el vínculo con la familia y su relación con los cuidados se enmarca en una sociedad que le ha asignado históricamente a dicha institución el rol de reproducción de la vida, siendo el ámbito doméstico el lugar principal para realizarlo. Si bien se nombran a diversos familiares, no son mencionadas las madres ni los padres. Esto se vuelve significativo, al entender que ambas personas son las figuras principales que plantea el sistema patriarcal en tanto organización de los cuidados de hijas e hijos.

Las narrativas también permiten advertir la tensión entre la importancia otorgada a la vincularidad familiar y el lugar efectivo en sus vidas. Por un lado, debido a diversas violencias ejercidas por parte de ciertos miembros de la familia y las marcas que ello imprime en sus trayectorias vitales. Por otro lado, debido a que no es posible aislar estas violencias, entendiéndolas como estructurales de un sistema patriarcal y desigual.

Los adolescentes se han encontrado “solos” (y/o con sus hermanas y hermanos), lo cual es posible pensarlo como estrategia de sostenimiento de las familias que, atravesadas por la precariedad, deben relegar la tarea de cuidados a sus hijas e hijos. Sin embargo, en esta asignación del rol se les otorga una responsabilidad que debiera ser de una persona adulta, y para la cual aún podrían no estar preparados. De esta manera no solo reproducen tareas y conductas que aún no han podido ser incorporadas en tanto aprendizaje, sino que se les quita la posibilidad de ser entendidos y tratados como niños. Al mismo tiempo, puede tener consecuencias no medibles en contextos donde los aprendizajes sobre cómo cuidar a otras personas están mediatizados por la reproducción de prácticas que pueden ser violentas y hostiles.

En otras ocasiones, encontrarse solos significa una salida o escape del hogar y de situaciones de graves vulneraciones y violencias. En este sentido, entendemos a “la familia como un espacio paradójico: es el lugar del afecto y la intimidad, pero es también el lugar privilegiado para el ejercicio de la violencia”^26^. Si bien desde el Estado se producen y refuerzan concepciones que ubican a la familia como la responsable de los cuidados cotidianos y de la reproducción de la vida, ello es escasamente acompañado en las prácticas.

Otro aspecto a destacar es que el valor de los vínculos familiares está ligado a ciertos gestos de cuidado en momentos específicos de sus trayectorias de vida. La presencia, el buen trato en algún momento, el afecto amoroso o el ser alojados en situaciones difíciles es aquello que vuelve significativo a estos lazos. Sin embargo, la importancia de estos vínculos no está dada por su periodicidad o por su constancia en los diversos momentos de su vida; sino que incluso aparecen en las narrativas desde recuerdos lejanos, episódicos y hasta construcciones explícitamente imaginarias.

Lejos de romantizar estas construcciones-otras, que no se dan sin graves vulneraciones y situaciones de padecimiento para los adolescentes, nos interesa repensarlas en tanto rearmar nuevos modos. Los adolescentes saben saltar roles, transitar espacios y al mismo tiempo ser echados de ellos y conquistar otros. Es decir, se puede ver en ellos la posibilidad de desandar aquello dado y reconstruir otros posibles^15^.

Contemplando las formas vinculares familiares que supieron sostener, una apuesta posible en tanto trabajadores del Estado es preguntarnos cómo acompañar el rearmado del tejido familiar en el contexto actual. Asimismo, cómo fortalecer en diversos espacios, personas adultas que se constituyan en referentes, ejerciendo roles asociados al cuidado, que puedan llevar a cabo prácticas subjetivantes que permitan el armado de otros posibles, en tanto poder construir otras formas o sentidos más amables y situados, que le retruquen a concepciones tradicionales de la familia.

#### Vínculo con La Boquita o el CAINA

La institución que ahora aloja a los adolescentes es algo más que las paredes que la sostienen o la política pública que pretendemos discutir. En los relatos, reaparece en diversas oportunidades la institución como un vínculo significativo en diferentes sentidos:

E: *¿Con qué personas te sentiste cuidado o que sentís que te cuidaron a lo largo de tu vida?*

Eze: *y bueno, ¡acá!* [...] *Lolo* [operador institucional] *de la mañana, hay veces que para que no me mande ninguna cagada el operador me marca.* (Entrevista 5, 2023)

*Al preguntar sobre qué espacios le parecía de cuidado Coco nombró a La Boquita.* (Registro de campo, 2023)

El vínculo con La Boquita o el CAINA se configura en diversas dimensiones: en tanto lugar físico, como espacio donde desarrollan actividades vitales y cotidianas como jugar, comer, dormir; por su dimensión simbólica, por lo que representa en tanto cuidado y resguardo y por experiencias previas habitando. Por el vínculo con las personas referentes adultas que la conforman, que son quienes están “ahí”, miran, abrazan o marcan un límite si se están por *“mandar una cagada”*.

Un motivo para que el vínculo con la institución tome estas formas, se relaciona con que las personas dependen de ámbitos de lo tangible y lo compartido, así como de una infraestructura que facilite la vida. Sin embargo, esta dependencia puede ser el escenario de la dominación o el encarcelamiento^27^. Lo diferencial en esto será la posición que tomen quienes habitan y hacen las instituciones:

*Pero acá* [La Boquita] *no es así, no te juzgan y te dejan volver a entrar para ver de qué manera cuidarte mejor*. (Registro de campo, 2023)

En sus narrativas resuena el haberse ido y luego regresar a la institución en reiteradas ocasiones. En sus relatos cotidianos, caracterizados por la descripción de la itinerancia y la circularidad en sus trayectorias, que exista un espacio físico que esté en el mismo lugar tiene un sentido positivo, en tanto poder ir, volver y encontrarlo. Más allá de los requisitos de entrada y salida, que sea posible-viable de algún modo el ingreso tiene sentido como un espacio al cual se puede volver y encontrar cierto resguardo y pausa.

A su vez, se advierte cómo la lógica de “lo inmediato” está presente en las narrativas, motivado por la satisfacción de necesidades. Lo diferencial en La Boquita y el CAINA es que aparecen posibilidades diversas y flexibles que alojan algo de los deseos, necesidades y la singularidad de cada inmediato. De esta manera, se habilita la construcción de otros tiempos, espacios y formas. Algunas acciones como irse cuando desean consumir o hacerlo en el interior de la institución, si bien tiene consecuencias, no necesariamente implican sanciones como quedarse afuera, perder la vacante o ser expulsado.

E: *¿y acá sentís que te tratan bien?*

Facu: *sí.*

E: *¿Cómo es alguien que trata bien?*

Facu: *No sé, que me hable bien, que me respete*. (Entrevista 1, 2023)

*Destaca a Aldana, Candela, y Valeria como las operadoras que lo cuidan y acompañan en el cotidiano. Aldana con temas de CV y búsqueda de trabajo, Candela con temas del colegio y Valeria (los fines de semana) salen a pasear, tomar helados, charlar. Dice que, para aguantarlo a él, tienen que tener mucha paciencia porque le gusta mucho hacer chistes y joder, y que él valora mucho a quienes si lo “soportan”*. (Registro de campo, 2023)

La existencia de estas instituciones, en tanto infraestructura, cobra un mayor sentido cuando son habitadas y construidas subjetivamente como espacio que los aloja de manera singular. El buen trato, que les hablen bien, que los respeten, que acompañen sus proyectos y deseos aparecen como aspectos necesarios que consolidan y fortalecen el vínculo con la institución. Son sus características las que hacen que las paredes signifiquen un interior situado y singular, diferente a otros espacios por los que transitan o transitaron.

Son las personas trabajadoras de la institución quienes, dentro de los límites y alcances de cada una, tienen la posibilidad de reorganizar y rearmar las dinámicas institucionales, las reglas y normativas. Es decir, la posibilidad de construir conjuntamente con los adolescentes estrategias situadas a las circunstancias que los atraviesan, en lugar de homogeneizar en nombre del deber, de lo esperado y de lo supuesto.

Es importante destacar que, en los relatos y encuentros colectivos con los adolescentes, también surgen aspectos que señalan y sobre los cuales se muestran en disconformidad.

*Al otro día cuando vuelven (de una salida) realizan un planteo a los operadores, diciéndoles que están todos a las corridas por otras situaciones y nadie los escucha.* (Registro de campo, 2023).

Los relatos dan cuenta que los vínculos construidos con referentes institucionales (operadores, equipo técnico, coordinadores) no se dan de manera homogénea, sino que existen tensiones y conflictos. Expresar aspectos con los cuales están en desacuerdo, da cuenta de la presencia de otra persona con la apertura y disponibilidad para recepcionar esa disconformidad y queja ante aquello que les genera malestar. En este sentido, sin haber enlazado previamente con otra persona, sin el afecto mediante o sin haber construido cierto pacto vincular mutuo, no sería posible el conflicto, ni el acuerdo.

E: *¿Y a dónde querés ir?*

Facu: *Quiero conocer otros pibes, a otros operadores.* (Entrevista 1, 2023)

Reconocer a las instituciones y sus diversos sentidos como parte constitutiva de sus vínculos implica, al mismo tiempo, dar cuenta de un continuo en la institucionalización de sus trayectorias de vida. Esa trayectoria se materializa en las narrativas y proyectos cuyo horizonte posible pareciera ser transitar a otra institución que permita un nuevo armado vincular. Con esto no pretendemos (des)valorizar este tipo de vínculo o el rol de la institución ya que, como desarrollamos anteriormente, quien los recibe es quien brinda cuidados y aloja; sino más bien preguntarnos nuevamente en tanto trabajadores de las diversas instituciones por las que circulan, ¿cómo co-construir otros horizontes posibles? O ¿cómo acompañar y fantasear sobre proyectos que tengan que ver con lo vivificante y no con un destino casi anunciado?

Asimismo, ser narrados constantemente por otras personas podría diluir las fronteras entre lo privado y lo público en las vidas de aquellos que habitan la calle y sus instituciones. Sus vidas, que suceden en la calle a la vista de todas y todos y, al mismo tiempo, de nadie; sus historias, puestas en debate constantemente, dejan poco lugar para aquello que desean que no sea narrado, contado. Ante esto, ¿cómo elaborar espacios que permitan construir pactos de intimidad que al mismo tiempo habiliten lo cuidado para narrar la propia historia?

En este sentido La Boquita y el CAINA aparecen como lugares donde confluir y encontrarse entre pares. Por lo tanto, muchas veces el vínculo entre ellos se entrama con el vínculo con la institución que los reúne, generando un escenario diferente al encontrarse en la calle, mediado por otras normas (explícitas e implícitas) y otra forma de circulación de los afectos.

#### Vínculo con pares/amigos

La referencia al vínculo entre pares no trata de una grupalidad homogénea y armónica. Por lo contrario, se caracteriza por la presencia de mutaciones, según los momentos y cotidianos que transitan. De este modo, no todos los pares con quienes coinciden en espacio y tiempo suelen ser amigos, sino que esta connotación aparece restringida a aquellos con quienes se comparten momentos de disfrute, a quienes aconsejan sobre cuidados y, en algunos momentos, marcan algunos límites o tienen que “bajar raya” cuando sienten que fueron incumplidos ciertos acuerdos:

Eze: *Bueno, mis amigos son medio boluditos, pero bueno, los quiero igual.*

E: *¿y por qué los querés igual?*

Eze: *y porque son buena onda. A veces cuando voy para el barrio hacemos rin raje*. (Entrevista 3, 2023)

E: *¿Cómo es la relación con tus compañeros?*

Bauti: *Y depende… hay veces que si no les bajo una raya se quieren abusar y… son muy caraduras.*

E: *¿Y cómo sería bajarles una raya?*

Bauti: *Yo les metería un bife, y si se para de manos, un par de piñas.*

E: *¿Y tenés amigos acá adentro?*

Bauti: *Sí… Rodri, el Mochi que se fue, el Lautaro…, pero al Lautaro le bajé una raya hoy también. Se quiso adueñar de mi visera, pero no lo dejé.*

E: *No sabía que Mochi se fue…, ¿lo extrañás un poquito?*

Bauti: *Sí, alto gato, al pedo se fue*. (Entrevista 3, 2023)

E: *¿Y si tuvieses algún amigo que se estuviera por mandar una cagada qué le dirías?*

Facu: *Que no lo haga.*

E: *¿Tenés que darle esos consejos a veces a tus amigos?*

Facu: *No, porque ellos ya lo saben, yo no tengo que decir, ya están grandes ellos*. (Entrevista 1, 2023)

Estos vínculos se constituyen desde la elección de otra persona por diversos motivos, que pueden ser desde lo compartido, por el mismo anclaje barrial o por haberse encontrado en La Boquita o el CAINA y haber construido ciertos pactos comunes.

En este sentido, se advierte la construcción de vínculos con pares desde el mundo del aguante. Los adolescentes eligen quienes son sus amigos en función de afinidades y distinguen entre compañeros de la institución y amigos a los cuales por momentos tienen que ponerles un límite; sin embargo, todos están atravesados por la lógica en donde prima cierta competencia masculina por mostrar valor, osadía y poder. Por lo tanto “*bajar una raya*” a alguien que se considera dentro de su grupo de amigos puede ser entendido como parte constitutiva de los vínculos que construyen y habitan. Esto puede suceder por la inscripción de códigos y normas propias, entre pares. Esta inscripción de códigos propios puede ser posible a partir de ritos de situación^28^. En este caso, el otro no se instituye a partir de la ley estatal o paterno-filial, sino a partir de las regulaciones grupales y de acuerdos propios.

Esta paridad y normas propias tienen otros bordes o tamices en situaciones que implican un cuidado. En este sentido, notamos

[la] presencia de discursos permeados por la diferencia generacional como distinción en torno a lugares fijos [...] construidos sobre el principio de autoridad y de saber: sabe el que ha vivido una experiencia y el que ha recibido la herencia acumulada.^28^

Aun cuando la diferencia etaria sea poca, se reproduce este principio:

Eze: *Fuimos a la casa de un amigo hace un mes, fuimos con unos chicos más y había un chico que le pegaban, 11 o 12 tendría y le pegaban. Yo me metí, nos metimos adentro y fue, entre cuatro y chau*.

E: *y ahí los cuidaste, ¡re importante eso!*

Eze: *no, me metí adentro para defender al pibito, llame a la policía…* (Entrevista 5, 2023).

*Le preguntamos a Samuel si tiene ganas de acompañarlo a Constitución y nos dice que no con la cabeza, lo mira a Mati y dice “pero él es chiquito”. Le respondemos que él también es chiquito. Samuel responde “¡naaa! bueno sí, pero yo entiendo, sé lo que significa”. Le preguntamos si va para cuidar a Mati y nos dice que sí*. (Registro de campo, 2023).

Si bien hay un cuidado entre pares, los gestos de cuidado son nombrados en las narrativas desde una posición de disparidad. Los amigos son para hacer o evitar “*cagadas*” mientras que a los más chicos se los cuida. Asimismo, ubica a quien ejerce estos cuidados en una jerarquía mayor con relación a quien lo recibe. Es decir, cuidar no solo es de los más grandes hacia los más chicos (aún en vínculos intrageneracionales) sino que también te hace más grande. Cuidar a un par no se narra como una posibilidad. En cambio, desde lo experiencial, circulan estrategias de cuidado y formas de vincularse con sus pares que responden a un reconocimiento igualador, como lo es marcar un límite, “*bajar una raya*” o “*rescatarse*”. Asimismo, entenderse como par implica una identidad y una serie de códigos propios, que a la vez dan cuenta de sus formas de habitar el mundo:

E: *¿Y qué crees que tiene que tener alguien para ser un amigo?*

Bauti: *Y no sé, respeto nomás, respeto y actitud.*

E: *¿Qué sería tener actitud?*

Bauti: *Y no sé, hay veces que se te quieren desconocer por una pelotudez y te da bronca eso.*

E: *¿Y qué hace que sea un poco más fácil vincularte o hacer amigos, llevarte bien con otros?*

Bauti: *no sé…* [silencio] *Que sea pillo, que no sea tan tarado de joder todo el tiempo, nada más*. (Entrevista 3, 2023)

“*Hacerla bien*”, “*ser o ponerse pillos*” podría remitir al uso de las propias estrategias y experiencias de habitar las calles y sus diversos circuitos. Al mismo tiempo, permean las relaciones de intercambio que se crean y construyen en los encuentros. Frente a una situación violenta no se invoca a la seguridad del Estado o sus instituciones, ponerse “*pillo*” permite el despliegue de modos individuales y colectivos de cuidado y protección. El que se “*pone pillo*” se coloca en una posición activa, haciendo gala de su astucia. Da cuenta de una economía de la atención barrial, de desplegar una sensibilidad que anuncia una situación difícil o violenta^15^.

### Estrategias de cuidado

Las estrategias de cuidado son enlazamientos de experiencias que los adolescentes transforman y moldean para cuidarse o cuidar a otros. Este andamiaje no es sin rupturas que (re)inventan y (re)escriben las historias y aprendizajes, en función de las necesidades singulares y situadas de cada momento. Se distinguen entre ellas el rescatar(se), el uso estratégico de instituciones y vínculos y el hablarle a otro (que se está por “*mandar una cagada*”).

#### Rescatar(se)

Entre las estrategias de cuidado que circulan resuena la posibilidad de rescatar(se), es decir, de poder salir o abandonar ciertas situaciones (límites) que podrían implicar un riesgo para su vida. Principalmente, apela a la posibilidad de construir o instalar un proyecto alternativo, que no se vincule a situaciones de consumos identificadas como problemáticas, en sus narrativas:

*Comenta que tiene ganas de alejarse de eso (en referencia al consumo), que está en la calle desde los 11 años y que él sabe que si sigue como estaba antes de entrar a la institución va a terminar o preso o muerto. Afirma que no hay otros lugares posibles, por eso decide estar ahí*. (Registro de campo, 2023)

*Ema le cuenta a Cala que está ahí por una medida de abrigo. Que se quiere rescatar y que no quiere estar en calle.* (Registro de campo, 2023)

Entonces, esta posibilidad de rescatar(se) pareciera anudarse a dos posibles. Por un lado, se enlaza el rescatarse con el poder o la responsabilidad individual. Es decir*,* “*nadie te va rescatar, vos ten*é*s que rescatarte solo*”. Si bien este enunciamiento responde a cierto deber ser moral, individualista y neoliberal, también permite desplegar ciertas estrategias conocidas y transmitidas para lograrlo. Por otro lado, en sus relatos, el rescatarse aparece como única opción para evitar un destino que se presenta como inamovible. Terminar “*preso o muerto*” como si fuera una continuidad en aquellas historias de adolescentes en las que sus vidas fueron criminalizadas o asesinadas por la violencia policial e institucional.

Al mismo tiempo, aparece otra posibilidad en relación con la estrategia del rescate: apelar a las instituciones y/o a las personas que las conforman para que “*te rescaten*”. Esto implica recorrer y transitar diversos espacios para encontrar a otra persona que pueda instaurar algún límite, enlazar o alojar algo que devenga rescate, al mismo tiempo habilitando otros posibles.

Sin embargo, rescatar(se) no es sin un costo o pérdida. Acarrea quedarse afuera de un grupo, dejar de ver a los amigos del barrio o la calle y dejar de contactarse por redes. Lucirse rescatado por el barrio o los pares de la calle aparece como una especie de abandono de una vida que se hacía en común a otros^15^.

A su vez, los vínculos que tienen que abandonar para rescatar(se) pueden ser tal vez los únicos presentes en ese momento de su vida. Entonces ¿qué otros lugares de construcción de vínculos se ofrecen para acompañar ese rescate? ¿Cómo promover el rescatar(se) cuando no hay otros lugares posibles o te quedás afuera?

#### Hacer uso estratégico de instituciones y/o vínculos

Hacer uso de su acervo de conocimientos construidos a lo largo de sus propias trayectorias, en pos de resolver alguna situación que están atravesando ellos o quienes quieren ayudar, se constituye como estrategia ante momentos específicos. Estos saberes incluyen los barrios por los que transitaron, las personas que los habitan y el conocimiento relacionado a los alcances y límites de las diversas instituciones:

Eze: *No, me metí adentro para defender al pibito* [al que estaban golpeando], *llamé a la policía, 50 patrulleros dentro de la casa, el chabón lo amenazaba, le conté todo a la policía cuando llamé, que el chabón estaba armado todo*…

E: *¡ah, re grave!*

Eze: *No, para que venga más rápido, ¿entendés?* [haciendo referencia a que agrandó la historia]. (Entrevista 5, 2023).

*Samuel recuerda que a veces dormía en el* [hospital] *Argerich, que una vez estuvo internado porque él lo pidió, también cuenta que un policía lo cuidaba, y que lo conocían ahí*. (Registro de campo, 2023)

Hacer un uso astuto de sus conocimientos sobre las instituciones y quienes las habitan, lejos de responder a lógicas ligadas a lo impulsivo o incluso al deber moral, permite pensar(se) en situación^29^*.* En la narrativa de los adolescentes se visibiliza que las estrategias son construidas de manera singular y situada reconociendo los límites y los alcances de las instituciones; es decir, cómo presentarse para obtener las respuestas esperadas, ante situaciones que no pueden esperar tiempos y protocolos institucionales.

Como se menciona anteriormente, “*ser o estar pillo*” no solo permea las relaciones de intercambio que se crean y se construyen en los encuentros, sino que implica el uso de estrategias y experiencias propias de habitar las calles y sus diversos circuitos.

El “*estar pillo*” se despliega como estrategia ante situaciones en las que la mera proclamación o enunciación de sus derechos se presenta como insuficiente e impotente, en este sentido mientras no puedan ejercer sus derechos en tanto ciudadanos, tendrán que crear y ensayar otras formas de cuidarse^15^.

Entendemos el uso estratégico de las instituciones como profanación, en tanto devolver el sentido común que es quitado^30^, y usar a la policía como defensa de quienes históricamente son violentados por dicha institución, como modo de trastocar la norma.

#### Hablarle a un otro (que se está por “mandar cagadas”)

“*No hacer cagadas*” o “*hacerla bien*” es parte de lo que permite la construcción y sostenimiento de vínculos que consideran significativos, lo cual se evidencia en los consejos que les brindan a sus amigos y/o pares. En este sentido los consejos no van direccionados a capturar cómo se debería o espera que se actúe según las normas institucionales, sino que están direccionados a promover ciertas condiciones de cuidado:

E: *¿y para cuidar a otros?*

Facu: *no sé, que no hagan lo mismo que yo, cagadas* (Entrevista 1, 2023).

E: *si un amigo se está por mandar una cagada, ¿qué consejo le darías?*

Bauti: *depende de la cagada… ¿Como por ejemplo fumar porro?*

E: *Ponele… o la de irse y no volver.*

Bauti: *Le digo que vuelva, que se fije… que la haga pero que le haga bien… Porque si la hace mal, no puede volver*. (Entrevista 3, 2023).

“*Hacerla bien*” algunas veces puede significar hacer uso de estrategias socializadas y compartidas por amigos y pares que atravesaron experiencias similares y que construyeron un saber relacionado a esas experiencias. Se hace alusión a consejos que posibilitan otro marco de acción, corriéndose de la normativa e incluso de la prohibición, como puede ser la del no consumo. En este sentido, el consejo no sanciona el “*fumar porro*” o “*irse*”, sino que abre otras condiciones de posibilidad que no son punitivas ni juzgan; por el contrario, pretenden disminuir las consecuencias de las acciones. Así, los consejos que se dan mutuamente podrían ser intentos de perdurar en los vínculos significativos, de crear otros modos de habitar esos lazos y reparar aquellos momentos en los que no hubo un otro para alojar. Esto podría estar dando cuenta de la inscripción de los cuidados en las redes de reciprocidad existente entre ellos:

*En ese momento Mati dice que va para Constitución (previamente nos dice que es para robar y gastarse la plata en “giladas”). Coco lo empieza a llamar, diciéndole que se quede*. (Registro de campo, 2023).

*Coco nos dice que cuando se quieren pelear entre compañeros, él intenta hablarles. Dice que es lo que hicieron con él, que cuando él entró también le hablaban y lo escuchaban de esa manera*. (Registro de campo, 2023)

Hablarle a un otro para no “*bardearla*” o “*bancar*” son otras formas posibles que toman las estrategias de cuidado. El cuidado conforma algo así como un intento de introducir una pausa. Hablar con quien se está por mandar una cagada, si bien alerta a que se rescate, pareciera que también intenta alojar desde la escucha y el entendimiento de lo que sucede. Nuevamente, se apela a reproducir aquello que tuvo efectos en la vida de cada uno, ahora con un otro.

## DISCUSIÓN

Como fue desarrollado, las dimensiones vinculares y las estrategias mencionadas se constituyen a partir de los sentidos atribuidos a los cuidados. Como se menciona al inicio, esta significación de los cuidados tiene que ver con las dimensiones subjetivas y materiales de cada persona. En particular, como se refiere en otros estudios^31^^,^^32^, los gestos de cuidado de estos adolescentes ocurren en contextos y situaciones de vulnerabilidad y violencias extremas que, al mismo tiempo, moldean estas formas de cuidar. El cuidado de lo propio (el cuerpo, lo material, los vínculos) se vuelve indisociable de las condiciones de vida, dado que en múltiples ocasiones el sostén de la vida depende exclusivamente de ellos. Es decir, los sentidos del cuidado se vinculan a la preservación básica de la sobrevivencia del cuerpo, que es amenazado por el contexto y el territorio hostil de la calle^31^. Asimismo, para estos adolescentes, el límite entre el espacio de lo público y el espacio privado, se vuelve borroso. Las estrategias de supervivencia, la diversión, el dormir, los vínculos sociales prioritarios, el consumo de sustancias psicoactivas o simplemente el “ser/estar” se desarrollan en el mismo espacio^31^.

Respecto a los vínculos afectivos, encontramos que responden (en ocasiones con ciertas contradicciones) a las definiciones de cuidado que ellos mencionan. En lo que respecta a la familia y, principalmente, a las personas significativas, se relacionan con vivencias específicas en sus trayectorias y/o a deseos de compartir más tiempo con ellos. Observamos que esto se repite en otras investigaciones que abordan situaciones de institucionalización de niñeces y adolescencias^33^^,^^34^, en las que integrantes de la familia se mencionan de manera significativa, a pesar de no compartir concretamente su cotidiano. Esto último se vuelve un interrogante sobre si realmente la periodicidad actual en esos vínculos es lo deseado o simplemente lo que esos referentes pueden brindarles. En este sentido, muchas veces circula que quien se encuentra habitando la calle no tiene vínculo con su familia. Sin embargo, esto puede suceder bajo formas y normas que se distancian de la “familia tradicional”, sobre la cual se asientan las representaciones sociales y el marco normativo actual.

Estas reflexiones ponen en tensión la privatización en el ámbito familiar de las tareas de cuidado. En este aspecto, tomamos los aportes de la concepción de los cuidados como derecho. Esto involucra de manera directa al Estado, desde un rol activo “independiente a los vínculos familiares y a las posibilidades económicas que existan en esos hogares”^35^.

En relación con La Boquita y el CAINA, encuentran aquello que definen como cuidado la o las personas que estén atentas a sus necesidades. En este sentido, se constituye como el espacio que posibilita enlazar y construir vínculos diversos, con tiempos, formas y afectos diferentes a los que se establecen en otros espacios. Estos aspectos marcan una diferencia en sus trayectorias de vida y de ello deviene la importancia del trabajo cotidiano que realizan en esta institución. Es decir, funcionan como contra-dispositivos^36^, entendidos como parte de movimientos instituyentes productores de modos de intervención que rompen con los modelos tutelares, disciplinarios y punitivos, y que se constituyen como puntos de fuga que promueven procesos de exigibilidad de derechos y de transformación. Este contexto se menciona como uno de los más significativos en experiencias similares^33^^,^^37^^,^^38^, que además se constituye como el principal sitio de apoyo por las relaciones interpersonales que entrama (tanto con personas adultas, como con pares) y se ponen en escena las amistades, los intercambios emocionales, las disputas y los conflictos. Entendemos que el mundo adulto habilita posibilidades para las trayectorias de vida de estos adolescentes^31^; por lo tanto, nos parece importante re-preguntarnos, como trabajadores del Estado, por la construcción de respuestas que acompañen circuitos vinculados al deseo y lo vivificantes, más allá del circuito calle/juzgado/hogar. Se trata de posibilitar formas de encuentro que “incluyan al otro en la trama discursiva institucional y social, permitiendo resignificar su propia historia”^31^.

Por otro lado, la elección del vínculo con pares y amigos se relaciona al mundo del “*aguante*” y al “*estar pillos*”. En esta línea, las características indispensables que definen a un amigo son, por ejemplo, el respeto y la lealtad, así como hacer las cosas bien vs la imprudencia^34^.

La elección fraterna es la dimensión que arma los lazos en el mundo del aguante y de la banda. La fraternidad [...] es una relación que no viene ya marcada por algún eje estructural, estatal o paterno-filial, sino que es puramente electiva. Se da por confianza, por saber que el otro se va a saber callar, que va a entender qué cosas se pueden decir y cuáles no, que se va a aguantar una apretada o que no va a traicionar.^28^

En este caso, el cuidado se presenta con contradicciones en lo verbalizado como tal y en lo concreto de sus actos (vivencias concretas que se materializan una serie de estrategias que dan cuenta de un cuidado mutuo).

Los bordes de estas estrategias de cuidado se rozan y dialogan constantemente. Como común, resuena la posibilidad de hacer uso de lo aprendido y compartido previamente, en momentos en que es necesario escapar o resolver una situación que pudiera implicar un riesgo (físico, material y/o vincular). En estas estrategias hay un “cuidado de sí” éticamente anterior al cuidado de otros^39^. El cuidado de sí implica inevitablemente la relación con otras personas en la medida que, para cuidar bien de sí, hay que escuchar consejos y/o experiencias.

Al remitir a la propia historia, el escape de situaciones riesgosas no significa un acto impulsivo; por lo contrario, se resuelve de manera singular y situada. Rescatar(se) y hacer un uso estratégico de las instituciones implica cierto despliegue de sus conocimientos. Ambas estrategias ponen en jaque las deficiencias del Estado y su política pública que no aloja de manera situada y concreta sus necesidades y sentires. Aún más, estas estrategias surgen porque aquello que debería acompañar sus procesos vitales finalmente los vulnera y los violenta.

Como tiempo medio de ambas, hablarle a quien se está por mandar una cagada pareciera instalar otro ritmo y forma. Si bien podría responder a cierta moral (“*no hacer cagadas*”) en línea con la posibilidad de rescatarse y hacer uso estratégico de las instituciones, podría inscribir un proyecto alternativo. En este sentido, “*hacerla bien*” permite otras formas no normativas, al mismo tiempo que habilita el traspaso de conocimientos y la creación de nuevas estrategias de cuidado.

De manera similar, en otras experiencias, estas estrategias aparecen mencionadas con puntos en común en la forma que toma ese cuidado. Un ejemplo de esto es un trabajo de investigación realizado con 146 niñeces y adolescencias^33^, en el que se mencionan categorías de apoyo, que podrían leerse como los efectos que generan las estrategias y gestos de cuidado. Estos tienen que ver con encontrar, en otra persona, apoyo emocional y afectivo; apoyo instrumental, vinculado a la ayuda material y de resolución de problemas; y apoyo relacionado al recibir un consejo o sentirse guiados.

Sus relatos dan cuenta de cotidianos e historias de vida atravesadas por lo fluctuante. Quienes están un día, pueden dejar de estar al siguiente, compañeros/amigos con quienes comparten la institución pueden decidir irse sin anticipar, al regresar al barrio que habitaron que pudo cambiar. Es la contingencia la que guía muchas de las acciones y vínculos construidos.

Lejos de romantizar lo difícil o inalcanzable que resulta en estos tiempos el armado de redes que sostengan y a las cuales se pueda volver, resaltamos la potencia y el valor de estos vínculos afectivos y las huellas subjetivas que imprimen, más allá de su itinerancia. Estos vínculos no están exentos de las estructuras sociales que nos atraviesan y que moldean nuestros cuerpos y nuestros modos de ser/estar en el mundo. Es decir, los vínculos que construimos están atravesados por el barrio en el que nos criamos, por la presencia o ausencia de políticas que acompañen, por las personas que fueron nuestras referentes, por los discursos que circulan sobre con quiénes podemos vincularnos o no, por los imaginarios sociales de quiénes somos o a qué grupo/s pertenecemos, por qué se considera una familia o no, entre otros. Y dentro de esta condición de posibilidad nos vinculamos con unas u otras personas en función de nuestras elecciones.

Las experiencias y estrategias compartidas dan cuenta de prácticas de cuidado vinculadas a formas que se (re)arman en los márgenes, de maneras más anónimas y/o populares. Formas-otras, no legitimadas en los términos del “buen cuidado” vinculado únicamente a la familia tradicional. Estas acciones o prácticas que surgen desde los barrios y desde la política pública (en tanto instituciones habitadas por personas que ponen en práctica acciones cotidianas) pueden ser pensadas como modos de producir cohesión con la comunidad y vínculos en contrasentido a la fragmentación de los lazos que imperan actualmente. Es decir, en sus diversas formas de vinculación y cuidado surge la posibilidad de construir puentes que permitan enlazar con un otre, diversificar vínculos y actores presentes en la cotidianidad, aunque éstos sean circunstanciales y cambiantes.

## CONCLUSIONES

A lo largo del trabajo de investigación pudimos poner en valor y en tensión otras formas vinculares. En este sentido, no desarrollamos sobre redes (en tanto red de vínculos de sostén y acompañamiento, a donde poder volver) ya que estas redes no aparecen en sus discursos. Si bien La Boquita y el CAINA parecieran ser lo más cercano a este tejido, una red no podría estar constituida por un único vínculo, sino que para categorizarla como tal debería coexistir con otros.

La elaboración sobre otras formas de armado de vínculos no fue sin contradicciones. Incluso bajo condiciones materiales y de existencia gravemente vulneradas, pudimos conocer y revalorizar las estrategias propias, los conocimientos y lecturas de los circuitos que transitan, en tanto sostén de esos vínculos.

Como referimos al inicio, nuestra intención fue poder conocer y elaborar sobre diversos sentidos y significados asociados al cuidado desde experiencias adolescentes. Fue parte de la discusión no solo establecer qué enlazaba los cuidados con los vínculos, sino determinar cuál entendíamos como constitutivo del otro. Acordamos que no hay una verticalidad rígida y delimitada en esta relación, ya que ambos dialogan y revisten de sentido al otro. Sin embargo, entendemos que en las narrativas el cuidado habilitaría la construcción de vínculos, así como las diversas formas que estos pueden tomar, según cómo sucedan los cuidados.

La itinerancia por diversos circuitos (del afecto, de la calle, en sus historias) se imprimen en las formas y tiempos que toman los vínculos que construyen. Aquellos significativos tienen que ver con cierta huella, cierto cuidado, con un momento compartido, con cierta complicidad o cierta palabra, sobre lo cual se vuelve, se rememora y se resignifica en la actualidad. Es decir, un amigo del barrio con el que jugaba o consumía, una abuela que alojó, algún lugar donde se habilitó fantasear, el compañero del hogar que pudo poner cierta pausa al consumo, una hermana o hermano que acompañó, una persona adulta que pude devolver una imagen diferente (y amable) de sí o un operador con quien se construyó algo diferente.

Las estrategias de cuidado de sí mismo o de otras personas entraman complejidades y sentidos que van más allá de ese acto concreto. Significan cierta continuidad de sus trayectorias por circuitos que supieron armar, que tienen que ver con lo vital y, al mismo tiempo, con padecimientos. Asimismo, enlazan los vínculos que cierto cuidado permite sostener (incluso, de manera más bien fugaz). Sin embargo, se plantea cierta contradicción, ya que aquello que las mantiene dentro de su circuito podría transformarse en su salida, si se elige rescatarse y si se habilitan los espacios para ello.

Retomamos el interrogante de cómo poder rescatarse, si pareciera no haber lugares para ello o si hacerlo significa la pérdida de vínculos significativos. Acordamos que dispositivos habitacionales (y vitales), como en este momento lo son La Boquita y el CAINA, son parte de una respuesta posible.

La elaboración de este escrito, las relecturas de las historias de vida y lo compartido con los adolescentes fue, al mismo tiempo, producción e intervención. Es decir, pudimos recuperar y resignificar sobre sus propias formas de cuidar, al mismo tiempo que esto implicó una serie de reflexiones sobre nuestras propias formas de acompañar trayectos vitales. Acordamos que co-construir espacios donde las adolescencias sean protagonistas permite co-crear estrategias que potencien los procesos de salud, poniendo en valor las experiencias de sus trayectorias de vida. Por otra parte, acordamos sobre la importancia de habi(li)tar espacios que permitan inventar, fantasear e imaginar sobre proyectos de vida deseantes, hasta el punto de que algo de eso devenga posible.

En el contexto actual, donde se vuelve a poner en debate la baja en la edad de imputabilidad, apostamos a políticas que alojen y no criminalicen a las adolescencias atravesadas por la precariedad; políticas públicas que reconozcan las actuales conformaciones y tramas podrían propiciar otras formas de crianza comunitarias, que acompañen y brinden espacios de cuidado a niñeces y adolescencias.

Apostamos a la continuidad de investigaciones dispuestas a co-crear propuestas con adolescencias, repensando las formas de construir conocimientos. De esta manera, no solo apelar a un lenguaje que resulte común y accesible para todas las personas, sino incorporar la palabra no hablada (gestos, expresiones, silencios) en los procesos de producción.
